# Research on Supply Chain Financial Risk Prevention Based on Machine Learning

**DOI:** 10.1155/2023/6531154

**Published:** 2023-03-06

**Authors:** Yang Lei, Hou Qiaoming, Zhao Tong

**Affiliations:** Shenyang University of Technology, School of Management, Shenyang 110000, China

## Abstract

Artificial intelligence (AI) proves decisive in today's rapidly developing society and is a motive force for the evolution of financial technology. As a subdivision of artificial intelligence research, machine learning (ML) algorithm is extensively used in all aspects of the daily operation and development of the supply chain. Using data mining, deductive reasoning, and other characteristics of machine learning algorithms can effectively help decision-makers of enterprises to make more scientific and reasonable decisions by using the existing financial index data. At present, globalization uncertainties such as COVID-19 are intensifying, and supply chain enterprises are facing bankruptcy risk. In the operation process, practical tools are needed to identify and opportunely respond to the threat in the supply chain operation promptly, predict the probability of business failure of enterprises, and take scientific and feasible measures to prevent a financial crisis in good season. Artificial intelligence decision-making technology can help traditional supply chains to transform into intelligent supply chains, realize smart management, and promote supply chain transformation and upgrading. By applying machine learning algorithms, the supply chain can not only identify potential risks in time and adopt scientific and feasible measures to deal with the crisis but also strengthen the connection and cooperation between different enterprises with the advantage of advanced technology to provide overall operation efficiency. On account of this, the paper puts forward an artificial intelligence-based corporate financial-risk-prevention (FRP) model, which includes four stages: data preprocessing, feature selection, feature classification, and parameter adjustment. Firstly, relevant financial index data are collected, and the quality of the selected data is raised through preprocessing; secondly, the chaotic grasshopper optimization algorithm (CGOA) is used to simulate the behavior of grasshoppers in nature to build a mathematical model, and the selected data sets are selected and optimized for features. Then, the support vector machine (SVM) performs classification processing on the quantitative data with reduced features. Empirical risk is calculated using the hinge loss function, and a regular operation is added to optimize the risk structure. Finally, slime mould algorithm (SMA) can optimize the process to improve the efficiency of SVM, making the algorithm more accurate and effective. In this study, Python is used to simulate the function of the corporate business finance risk prevention model. The experimental results show that the CGOA-SVM-SMA algorithm proposed in this paper achieves good results. After calculation, it is found that the prediction and decision-making capabilities are good and better than other comparative models, which can effectively help supply chain enterprises to prevent financial risks.

## 1. Introduction

The operation and development of an enterprise have been a new topic for a long time, but it is inevitable that an enterprise will inevitably encounter financial risks during its process. Risk prevention of an enterprise is a hot topic among many scholars. The 2022 government work report also repeatedly mentions the risk prevention of enterprises in its overall requirements and development orientation for the development of economic and social. Against the backdrop of the normalization of the COVID-19 epidemic, China successively issued a series of credit policies, tax reduction and fee reduction policies, and tax retention policies for preferential farmers and preferential enterprises. The relatively loose monetary policy provided a stable guarantee for the continuous operation of enterprises. However, due to the widespread COVID-19 and the instability of the global economy, many supply chain enterprises are still struggling and facing the crisis of bankruptcy crisis. Bloomberg data show that in 2021 there were 121 large-scale bankruptcy cases with debts of more than USD 50 million. It can be seen that the turmoil of the world economic situation and the erosion of the COVID-19 epidemic has brought many challenges to the daily operation of enterprises. Supply chain financial risks will not only harm the operation status of enterprises but making it difficult for enterprises to move forward in the operation process. Enterprises will be cautious in their investment and financing decisions, and enterprises with more significant financial risks will even face hidden dangers such as bankruptcy liquidation. The financial and financial status of supply chain enterprises is a support for the operation, and an excellent economic situation is a premise for the sustainable development of enterprises. Financial risks will seriously damage the financial indicators of enterprises. Therefore, it is imperative for the supply chain must take measures to reduce financial risks and strengthen financial risk prevention.

Affected by global debt and the COVID-19 epidemic, many supply chain enterprises cannot make ends meet and seek financial support from social and financial institutions. For minor and moderate-sized enterprises at the tail terminal of the supply chain, it is hard for banks to assess the credit risk of enterprises due to their imperfect management system and capital structure when facing the financing problem. Although banks will collect the soft data of enterprises on time to deal with the shortage of credit data, due to the lack of transparency, timeliness, and accuracy of credit data, banks will require higher financing costs from financing enterprises to reduce their own loan risk, which undoubtedly increases the financing burden for enterprises facing the financing problem and increases the financial market risk faced by enterprises. For the core business of the supply chain business, due to their vast operation system and enormous demand for capital, they are facing more significant financial risks such as operational risk, credit risk, and settlement risk. Banks will be more stringent in assessing the credit risk of enterprises, and the assessment will be more comprehensive and accurate. Therefore, enterprises should strengthen their efforts to guard against potential financial risks. Under the background that the business crisis has an essential influence on the global economy, it is an important research topic to accurately calculate the financial risks faced by enterprises and to explore ways to prevent digital financial risks in supply chains.

In the process of operation, the supply chain will face a series of risks, such as market competition risk, the credit risk of trade credit, national related policy risk, national legal risk, manager behavior risk, stakeholder information transmission risk, and natural environment risk. All kinds of threats will harm the operation of the enterprise. The supply chain should avoid these risks as much as possible when making decisions. Supply chain internal managers will be affected by factors such as market uncertainty, debtors' delay in paying debts, changes in their credit ratings affect the amount of financing, and changes in exchange rates resulting in poor foreign exchange translation when making operational decisions, which will cause certain risks to the property operation of enterprises. During the operation process of corporate, the digital financial risks faced by enterprises will also be exacerbated by the disagreement of the directorate, conflicts between the interests of stakeholders and the benefit of the enterprises, agency problems of senior managers, inefficient investment, and other issues. Due to the diversity and variability of financial risk types, it is difficult for managers to make scientific and reasonable decisions based on personal will. Financial risk prevention (FRP) model has essential significance for supply chain enterprises to make financial decisions. The model can consider various objectives of supply chain operation, make use of its advantages of data mining and data processing, visualize all kinds of financial data, facilitate managers to make decision analysis based on relevant data, and help leaders to make the best decision. Especially in today's significant data era, we should collect and sort out masses of data with the help of relevant models and get the utmost out of the preponderance of big data to help supply chain enterprises make reasonable decisions and promote long-term development.

At present, artificial intelligence (AI) technology has already been extensively used in diverse fields. Using machine learning algorithms can rapidly and availably arrange data and make decisions. The model constructed by machine learning algorithms can arrange myriad kinds of matter in supply chain enterprises, such as sales forecast, marketing strategy management, customer support, and order management. The most applied fields are the media field and the retail field. This paper uses machine learning algorithms to dispose of the financial risk, measures the potential risks faced by supply chain enterprises, helps enterprises make reasonable decisions, and improves the decision-making quality of managers. Collecting and collating the data of the quoted company in the supply chain of China in recent three years, preprocessing the collected data to upgrade the quality, effectively reflecting the operating conditions of all aspects of the enterprise, helping to apply the model to identify potential financial risks faced by enterprises, and improving the supply chain management capability. The chaotic grasshopper optimization algorithm can draw the economic characteristic from the selected listed companies' financial statements, and the support vector machine algorithm is used to classify the data after feature selection. For the sake of raising the precision of data classification, the slime mould algorithm is designed to further process and make the indicators more optimized after data classification, on purpose to enhance the demonstration of the decision-making system. According to the relevant standards, the financial risks that an enterprise may face are classified, and different early warning standards are set, which is helpful for managers to make decisions based on various data parameters, timely discover the risks faced by the enterprise, and take corresponding management measures to prevent hazards.

During the operation of the financial risk prevention model (FRP), feature selection (FS) is an essential stage in the model, which is mainly to filter out the critical and irregular features needed for decision-making from broad-minded data. The feature extraction module can be segmented into dependency filter type, wrapper type, and embedded type according to the evaluation status. Among them, packaging will face the problems of maximum processing complexity and learner constraints, while the implementing of embedded is more complicated. In contrast, the dependency filter can calculate the feature subset by using the permanent value and then detect the optimal feature from the accessible features. This method has significant advantages. Secondly, in the data classification module, the hackneyed categorize algorithms are support vector machine, K- nearest neighbor, decision tree, etc. The K- nearest neighbor algorithm needs to traverse all the data when classifying the data, which has poor timeliness, is sensitive to the K value and greatly influences on the precision of the ultimate policy decision. The decision tree algorithm is prone to overfitting, especially when there are more data, and the probability of the error rate will increase faster. In the data with a substantial degree of association, the algorithm cannot achieve good results. The support vector machine algorithm can simplify the classification process based on the existing training samples, which can have a good improvement on the efficiency and quality of classification. This method is not sensitive to the selection of kernels. It can facilitate the problematic position of multiclassification and multidimensional sample space by constructing the association of support vector machine and has certain robustness. Therefore, compared with the K- nearest neighbor and decision tree methods, support vector machine is more intuitive and accurate in the classifying of parameters and has certain advantages. In this model, the slime mould algorithm can further optimize the classification results and enhance the efficiency and practicality of the consequence. The financial risk prevention model can perform complex operations of checking economic data and related data, involving production accounting of enterprises, process of working capital, strategic development of organizations, and other aspects. Managers can improve their decision-making quality according to the relevant index parameters of the model, better manage the financial risks of the company and strengthen risk prevention.

When current scholars study the prevention of supply chain financial risks, some scholars expound the factors that affect digital financial risks from the angle of empirical analysis and put forward the management and countermeasures to prevent risks; some scholars use machine learning algorithms to analyze supply chain financial risks, such as neural network algorithm and TOPSIS method; at present, many scholars use a single machine learning algorithm to solve supply chain financial risks. In this study, a combined optimization method is proposed. CGOA-SVM-SMA algorithm can be used to optimize the relevant results. Compared with the previous research, it can better enhance the practicality of the pattern. The financial-risk-prevention (FRP) model depends on the artificial intelligence algorithm proposed in the essay and principally contains four stages: data preprocessing, select features, classification, and parameter adjustment. First, collect various financial data of the enterprise and preprocess them to enhance the weight of the selected data; secondly, chaotic grasshopper optimization algorithm (CGOA) can choose features and optimize the selection process. Then, support vector machine (SVM) algorithm can classify the data with reduced features, and the classification efficiency can be optimized by slime mould algorithm (SMA), to augment the accuracy of the final decision. Finally, tests are executed on data sets, and adjustments are made continuously to enhance the expression of the model's prediction decisions. The main innovations of this essay include the following theories:

1. Innovatively put forward a model to help supply chain enterprises guard against financial risks, using the CGOA-SVM-SMA algorithm, which mainly includes the feature selection of CGOA, classification of SVM, and parameter adjustment of SMA.
2. The CGOA algorithm is proposed for feature selection and optimization, which can effectively drop the sophistication of data calculation and enhance the prediction function of the pattern. The algorithm is applied to the novel hinterland of supply chain finance.
3. The slime mold algorithm can refiner the classification process of the pattern to enhance the management effect and decision-making performance of the pattern, and the innovative algorithm is used to refine the classification process to improve the efficiency.
4. The algorithm of SMA-SVM is proposed in the classification process, in which the SVM is used to classify and sort the data after feature selection. Then, the SMA is used to optimize these data, to strengthen the usefulness of the model. The former literature seldom uses SMA to enhance the specifications of the SVM, and the new model suggested in this study can significantly enhance the catalog efficiency and accuracy;
5. The rapid development of financial technology makes supply chain digital finance face unprecedented risks. The degree of integration between financial technology and supply chain finance in previous studies is not high. The innovative digital financial risk prevention model constructed in this study can help supply chain enterprises to be timely perceive potential risks in the Internet era with the advantages of financial technology. The model has high accuracy in prediction and decision-making and has specific practical value.
6. The machine learning algorithm is applied to supply chain financial risk management. This paper breaks the previous single research method, explores the machine learning algorithm combination optimization model, solves the management problem of the actual operation of the enterprise, and constructs the enterprise risk prevention and early warning mechanism, which helps the enterprise to prevent financial risks, enriches the application scope of the machine learning model, and solves practical problems.

Based on the above-given analysis, the overall organization of the essay is as follows. Because of the present situation of introducing the research background of this paper in Section 1; Section 2 introduces the current work related to financial risk prevention, as the research basis of this paper, which is helpful for further analysis in the light of the current research in the following part; next, Section 3 constructs the financial risk prevention model and elaborates the model in detail; In Section 4, relevant data are selected to verify the proposed model, which proves the feasibility of the model. Finally, Section 5 is a summary and outlook, which summarizes the paper and points out the research localization and the research field in the future.

## 2. Related Work

Economic globalization has increased communication among countries, making capital markets more active. With the increase in the amount of capital in circulation, the capital requirement of enterprises is augmenting increasingly. But in the meantime, it has also expanded the impact of adverse events such as the COVID-19 epidemic, causing turmoil in supply chain financial markets. Enterprises are in urgent need of financial risk prevention measures to deal with the impact of globalization.

### 2.1. Evaluation of Supply Chain Digital Financial Risk Prevention System

Using the advantages of AI and ML algorithms, a financial risk prevention model is built to study the influential risk factors affecting the operation of supply chain enterprises, and a judge target setup of enterprise monetary affairs risk is constructed, which can efficaciously heighten the prediction accuracy and decision-making correctness [1]. In the territory of finance of supply chain, with the development of the digital economy, the application of digital currency is more and more extensive, and its impact on the social economy is also more prominent. This emerging digital currency has gradually exposed its risks as it continues to grow [2]. To accurately predict the digital decision-making currency and reduce its operational risk, an improved deep neural network algorithm (DNN) is adopted, which uses information such as transactions and monetary returns to extract the relevant characteristics of Bitcoin to estimate and predict the price of Bitcoin, thus contributing to better e-commerce decision-making and reducing financial risk [3].

Machine learning algorithm has now been proverbially utilized in various domains. Applying it to the supply chain finance field can help realize the changing and escalating of business intelligence, which meets the requirements of the industry 4.0 era. Financial risk is a significant problem faced by many supply chain enterprises in the current period, which also affects social and economic development [4]. Applying the ML algorithm to the financial risk management of companies can get the utmost out of the advantages of the machine learning algorithm to solve the decision-making problems facing the supply chain. The machine learning algorithms can manage market risk, credit risk, etc. However, it is currently facing challenges in data, algorithms, and models in the application process. It needs to continuously optimize its algorithm to solve practical problems [5]. Some scholars conducted a systematic review of 232 studies, analyzed the necessity and urgency of applying artificial intelligence technology to identify the financial difficulties of supply chains, and pointed out that in data preprocessing, attention should be paid to data balance and dimensionality reduction, and the evolution index should be optimized to improve the model performance [6]. Some scholars also apply the machine learning algorithm to the operational risk management faced by the enterprises in the operation course. In manufacturing enterprises, the machine learning algorithm can diagnose the thermal imaging fault of brushless DC motor ventilation in enterprises, to improve the business's ability to resist the operation risk [7]. Aiming at the operation failure problem of electric percussion drills in the supply chain of manufacturing enterprises, the binary image difference common area (BCAoID) method is used for feature selection. Then, the nearest neighbor classifier and back propagation neural network are used to analyze the data after feature extraction. It is found that this method has good accuracy, can effectively detect the failure problems faced by enterprises in the process of application, and can help enterprises to make early decisions on the method risk [8].

### 2.2. Research Progress on Prevention of Digital Financial Risk in Supply Chain

With the universal of informative Internet science and technology, the management model of “Internet plus Finance” is requisite. It uses the advantages of advanced technologies such as mobile communication and blockchain to solve the financial management problems in the supply chain, strengthen the monetary affairs administrator in the operation process of enterprises, and decrease the economic affairs risks. To evaluate the threats brought by transactional finance in the supply chain, a machine learning algorithm can form a matrix for forecast analysis [5]. The average merge method can establish a blend dominate mold, and decision-making is made using algorithms such as the decision tree model, gradient promotion model, and random forest model. The operation status of the enterprise is analyzed, and risk prediction indicators are proposed to continuously optimize the demonstration of the risk dominant models, to raise the natural capacity of the enterprise to resist monetary affairs risks [9]. For the network credit financial risks in the supply chain, a corresponding risk early warning mechanism should be established. The P2P peer-to-peer lending model is a typical model of the Internet finance industry [10]. Analyzing and researching its risks is helpful to find out the risk factors in the whole of Internet finance. The weighted KNN algorithm lies in volatile accuracy, and the coarseness set can control the Internet financial risk. Using the relative superiority and inferiority resembling notion of diverse accuracy and coarseness set, different training sets are divided into a central region and boundary region. According to the matching of the sample center and test sample, the area to which the selection belongs is obtained, and the category of the part to which the instance belongs is determined. At the same time, the risk level of each sample can be determined by a quantitative weighted KNN algorithm, which is beneficial for managers to make scientific and reasonable decisions [11]. For the financial risks of capital operation in the supply chain, it can also be designed by using the web application program in the network embedded system. Using the embedded design, it can realize the online operation risk control and prompt user behavior, embed the risk detection system of the financial software module in the daily decision-making process, predict the risks of market risks, trade credit risks, Internet risks, liquidity risks, etc., establish an early warning mechanism, and construct a multimonitoring and discriminant analysis model for risk early warning [12]. Following the requirements of relevant accounting standards, continuously improve the Internet accounting control system, track and warn online payment fees and accounting efficiency in real-time, control accounts receivable ratio, shorten collection aging, improve fund management efficiency, improve risk prevention capability, and control financial risks of enterprises [13].

For credit card financial risk in the supply chain, some scholars use an artificial neural network to detect credit card fraud risk [14] and use supervised machine learning technology to classify credit card transaction data into fraud cases and accurate cases. Using an artificial neural network to detect credit card fraud as a binary classification task, the digit set is segmented into 315 legal transactions and 492 fraudulent transactions. The accuracy of the trained model can reach 99.95%. The artificial neural network model can well predict credit card fraud transactions, help managers make decisions in advance, and prevent financial risks [15]. There are also some relevant scholars who use the differential evolution superparameter optimization method to detect credit card fraud, use the differential evolution algorithm to deal with the data imbalance problem, and use the optimized XG-Boost algorithm to classify fraudulent transactions. The model has high accuracy after evaluation [16]. Some scholars also analyze the detection performance of fraudulent transactions based on a meta-heuristic algorithm and use meta-heuristic technology to optimize the superparameters, which can validly enhance the usefulness of fraud examination systems, simplify the detection process, and shorten the detection time [17]. The genetic algorithms can also refine the super specifications of fraudulent transactions, and genetic algorithms and network search algorithms are compared and analyzed to compare the accuracy of credit card fraudulent transactions experiments. The practical consequence shows that the genetic algorithm has higher prediction precision and better performance than decision tree, logistic regression, random forest, and other algorithms [18]. In addition, the particle swarm optimization algorithm can also refine the superparameters of the deep neural network to check credit card swindle trade. In contrast with the network search algorithm, the particle swarm optimization algorithm has better performance in precision, accuracy, recall rate, and other aspects, which can simplify the decision-making time and heighten the usefulness and effectiveness of fraud transaction detection [19].

As for the financial risks of bank loans, financial institutions are vulnerable to fraudsters and are exposed to fraud risks. Artificial intelligence technology helps to collect and sort out data, collect and evaluate credit information of lenders, etc. It can reduce the risk of default of lenders faced by banks [20]. Still, too excessive risk prevention will also cause some potential beneficiaries to lose the chance of loans, which is not conducive to the popularizing of national fiscal policies. Therefore, the risk prevention coefficient of bank loans should be moderate, and the efficiency of loan management needs to be improved urgently [21]. Most banks adopt a labor-intensive management approach, but it is less effective and will cause many people to lose their jobs. The traditional management approach has been unable to fully satisfy the requirement of monetary affairs organizations fully. It obliges make use of artificial intelligence, machine learning, and other models to enhance the risk prevention capabilities of financial institutions and improve their governance level [22]. Artificial neural network is used to examine the swindle risk in bank advance administration, which not only restricts the behavior of loan beneficiaries but also monitors loan fraud. Selecting the loan credit data of more than 600 customers of a bank, extracting the features, and then predicting and making decisions, the precision of the mold can achieve 98%. This model can effectively help financial service institutions to detect credit risk and prevent the harm caused by fraud [23].

For supply chains in different industries, machine learning algorithms can also solve the financial risk problem of business [24]. For the energy supply chain, we can not limit ourselves to the traditional regression method [25] but use a meta-elastic network learner to constitute the data of various learners, to achieve the purpose of prediction and decision-making. By adding additional energy prediction indicators for the industry to the decision-making data, potential risk information faced by enterprises can be predicted more pertinently, and the accuracy of the final decision can be improved [26]. For the supply chain of the construction company, it is particularly imperative to distinguish the risks of the enterprise and take corresponding measures to control various hazards [27]. Cross-analysis-machine learning model can be used to predict the chances of large-scale construction projects in the construction industry. By obtaining from the company the construction cost, completion time, quality control, impact scope, and other factors that will affect the operation risk of the enterprise of large-scale construction projects, and obtaining from the experts the Likert scale after scoring each risk factor, the received data are further processed, mainly for the identification of high-risk components and relevant low-risk elements, and performing K-means clustering analysis to get features with apparent characteristics. Firstly, descriptive statistics are carried out to analyze each data type; then, a few excessive-sampling skills (SMOTE) and Wilcoxon rank and sum test are synthesized, which helps to retain essential eigenvectors such as construction cost, completion time, and quality control. Finally, the genetic algorithm-based K-means clustering analysis algorithm (GA–K-means) employs the biobjective gamma to break up the extreme risk elements and the related low-risk factors, thus helping companies to determine different risk levels and take corresponding measures promptly [28].

## 3. Financial Risk Prevention Model

**Figure 1:**
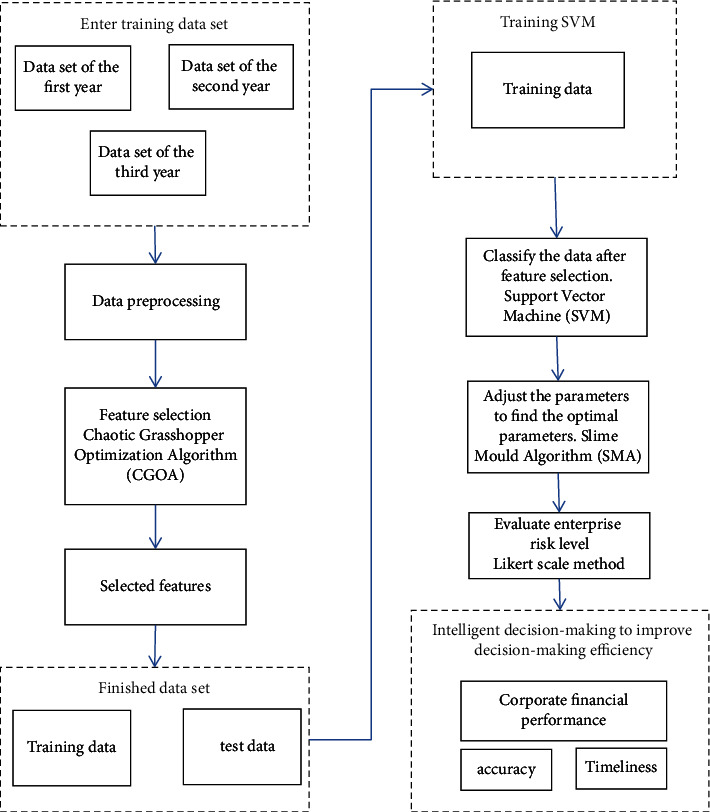
Workflow of enterprise financial risk prevention model.

As can be seen from the relevant research foundation, for financial risk prevention, most scholars use the neural network algorithm to study the relevant content, and few scholars apply the chaotic grasshopper algorithm to the supply chain financial risk prevention model and introduce the slime mold optimization algorithm to optimize the superparameters in the classification process. The workflow of the algorithm of the supply chain digital financial risk prevention model suggested is shown in Figure 1. First, collect relevant financial data of supply chain enterprises and input these data into the training set as training data; secondly, the data are preprocessed, and the CGOA algorithm is selected to select the features of these data. Putting the chosen functions into the training data set, and establishing a part of test data to confirm the precision of the pattern at a later stage; then, the classification process is carried out, SMA is used for parameter adjustment, and the SVM algorithm is used for data classification. Finally, these data are added to the trained model, and the precision of the mold operation is evaluated.

### 3.1. CGOA Based on Feature Selection

Grasshopper optimization algorithm (GOA) was initially proposed by Saremi et al. [29] in 2017, and Scholars Fouad [30], Ukasik [31], and Meraihi et al. [32] improved the algorithm, respectively. As a meta-heuristic bionics optimization algorithm, its search usefulness and convergence capability are better. Its unique adaptive mechanism can better bring into equilibrium the general situation and local search process, thus improving the search accuracy. The locust optimization algorithm simulates the removing and finding foodstuff behavior of the locust population in the natural world and divides the whole process into an exploration process and a development process [33]. The algorithm is principally a compound of three factors, and its scheme process is shown in the following formula:

(1) $$\begin{array}{c} X_{i}=S_{i}+G_{i}+A_{i}\text{.} \end{array}$$

Here, *X*_*i*_ indicates the position of the *i*-th locust, *S*_*i*_ expresses each other attraction between individual operations, *G*_*i*_ indicates the gravitational force of the *i*-th locus, and *A*_*i*_ means the wind intensity received by the *i*-th locust. The location of locusts is most affected by social action, and the formula for calculating *S*_*i*_ is

(2) $$\begin{array}{c} S_{i}=\overset{N}{\underset{\begin{array}{c} j=1\\ j\neq i \end{array}}{\sum }}S\left(\left|X_{j}-X_{i}\right|\right)\frac{X_{j}-X_{i}}{d_{ij}}\text{.} \end{array}$$

Here, *N* represents the total quantity of locusts in the community, *d*_*ij*_ indicates the range between locust *i* and locust *j*, and *S* is the function of social strength. The estimation process is as follows:

(3) $$\begin{array}{c} S\left(r\right)=fe^{-r/l}-e^{-r}\text{.} \end{array}$$

Here, *f* and *l* are the social attractiveness strength and social attractiveness scale, respectively, and *r* represents the actual social value of its existence.

Secondly, the formulas of *G*_*i*_ and *A*_*i*_ are

(4) $$\begin{array}{c} G_{i}=-ge_{g}\text{,}\\ A_{i}=ue_{w}\text{.} \end{array}$$

Here, *g* is the gravitational force target, *e*_*g*_ is the normalized vector facing the earth, *u* is the wind literal, and *e*_*w*_ is the normalized vector direct to the direction of the wind. Therefore, the position *X*_*i*_ of the locust can be expressed as

(5) $$\begin{array}{c} X_{i}=\overset{N}{\underset{\begin{array}{c} j=1\\ j\neq i \end{array}}{\sum }}S\left(\left|X_{j}-X_{i}\right|\right)\frac{X_{j}-X_{i}}{d_{ij}}-ge_{g}+ue_{w}\text{.} \end{array}$$

However, in this mathematical model, the locusts will quickly find a comfortable position, and the population will not converge to a specific value, requiring optimization. At this time, the gravity factor *G*_*i*_ is not considered, and the wind direction always points to *T*_*d*_, so the improved mathematical model after optimization is

(6) $$\begin{array}{c} X_{i}=c\left[\overset{N}{\underset{\begin{array}{c} j=1\\ j\neq i \end{array}}{\sum }}c{ub}_{d}-{lb}_{d}/2s\left(\left|x_{j}-x_{i}\right|\right)x_{j}-x_{i}/d_{ij}\right]+T_{d}\text{.} \end{array}$$

Here, *ub*_*d*_ and *lb*_*d*_ are the superior and inferior bounds of S(r) in the d-dimensional room, individually, and *T*_*d*_ is the optimal solution of the locust location in the d-dimensional room. *c* is the decreasing coefficient, and the calculation formula is

(7) $$\begin{array}{c} c=c_{\max}-n\frac{c_{\max}-c_{\min}}{n_{\max}}\text{.} \end{array}$$

Here, *c*_max_ and *c*_min_ are the biggest and smallest number of parameters *c*; individually, *n* is the times of iterations present, and *n*_max_ is the biggest times of iterations.

The population initialization of the traditional GOA algorithm is entirely random, which cannot guarantee the ergodicity of the population in the sample space, and the optimization accuracy is poor. Therefore, this paper introduces the initialization method of chaotic mapping on this basis, using Tent mapping to obtain [0, 1] constant value in the space. Its expression is

(8) $$\begin{array}{c} y_{i,j+1}=\left\{\begin{array}{cc} y_{i,j}/u\text{,} & 0\leq y_{i,j}<u\text{,}\\ \left(1-y_{i,j}\right)/\left(1-u\right)\text{,} & u\leq y_{i,j}<1\text{.} \end{array}\right. \end{array}$$

In the formulation, *i* is the population size, *i*=1,2, ⋯*N*, *j*=1,2, ⋯*d* , and *j* is the chaotic serial number, which represents the spatial dimension of the individual. Take the random number of [0, 1], determine the chaotic parameter of [0, 2], take the initial value of formula (8) to obtain the *y* chaotic sequence *y*_*i*,*j*_, and then use formula (9) to perform chaotic mapping:

(9) $$\begin{array}{c} x_{i,j}=l_{j,\min}+y_{i,j}\times \left(u_{j,\max}-l_{j,\min}\right)\text{.} \end{array}$$

In the formula, [*l*_*j*,min_, *u*_*j*,max_] represents the search range of *x*_*i*,*j*_.

The adjustment formula for parameter *c* is

(10) $$\begin{array}{c} c\left(l\right)=\left\{\begin{array}{cc} c_{\max}-\left(c_{\max}-c_{\min}\right)\times \frac{1+{\left[\cos l/L\pi \right]}^{2}}{2}\text{,} & l\leq \frac{L}{2}\text{,}\\ c_{\max}-\left(c_{\max}-c_{\min}\right)\times \frac{1+{\left|\cos l/L\pi \right|}^{2}}{2}\text{,} & \frac{L}{2}<l\leq L\text{.} \end{array}\right. \end{array}$$

In the formula, *l* and *L* are the times of iterations present and the biggest times of iterations, individually and *c* shows nonlinear iterative decrement. In the initial phase of iteration, *c* is enormous, and the decrement velocity is sluggish, which can seek the population data; in the posterior period, *c* is small, and the decline rate is faster, so it can quickly converge with good convergence effect.

In addition, CGOA takes into account the leading role of elite individuals *T*_*d*_′ ^∧^′ on the position of the population in the iterative process and introduces a probabilistic perturbation strategy to the current elite individuals, and the perturbation probability *P*_dis_ is

(11) $$\begin{array}{c} P_{dis}=\left(d-1\right)e^{\left(l-1\right)/L}\times /4d\text{.} \end{array}$$

The perturbation method is stochastic and produces a random digit *r* between [0, 1]. If *r* > *P*_dis_, individual perturbation is required. If *r* ≤ *P*_dis_, the original individual remains unchanged.

At the same time, the Cauchy variation mechanism is introduced. The Cauchy distribution is uninterrupted, and it represents probability [34]. The probability density function of the one-dimensional is

(12) $$\begin{array}{c} f\left(x\right)=\frac{1}{\pi \left(x^{2}+1\right)},x\in \left[-\infty ,+\infty \right]\text{.} \end{array}$$

Introducing the Cauchy operator into the position formula of the current optimal solution, we obtain the following equation:

(13) $$\begin{array}{c} X_{new}=cauchy⊗X_{best}\text{.} \end{array}$$

In the formula, Cauchy is the Cauchy operator obeying the Cauchy distribution.

Combined with the disturbance probability, the current optimal position is

(14) $$\begin{array}{c} X_{new}=\left\{\begin{array}{cc} cauchy⊗X_{best}\text{,} & r>P_{dis}andF\left(X_{new}\right)>F\left(X_{best}\right)\text{,}\\ X_{best}\text{,} & r\leq P_{dis}andF\left(X_{new}\right)\leq F\left(X_{best}\right)\text{.} \end{array}\right. \end{array}$$

**Figure 2:**
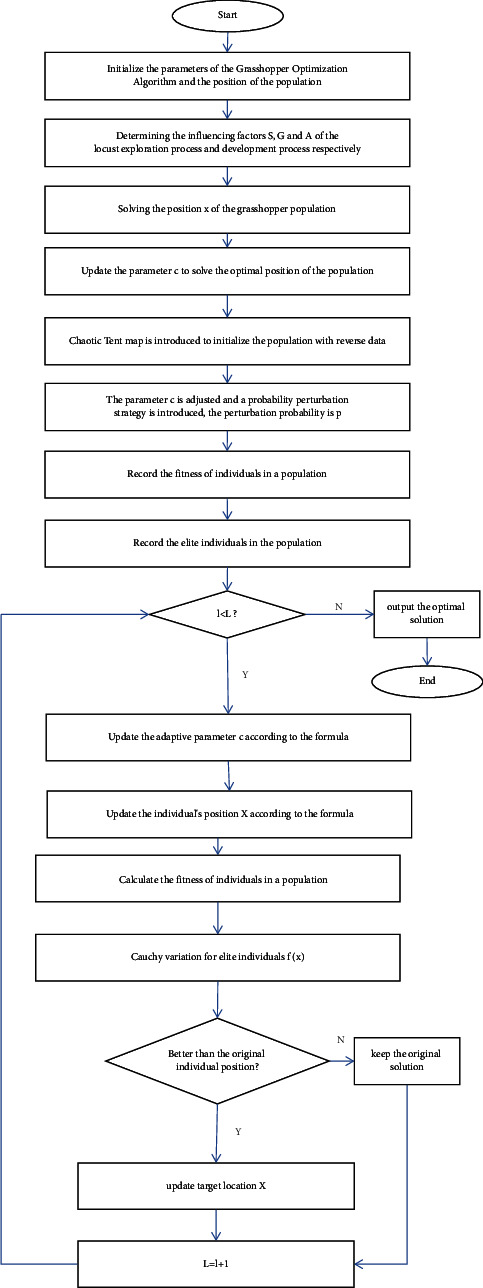
The execution flow of the CGOA algorithm.

In short, the overall execution process of the CGOA algorithm is revealed in Figure 2.

CGOA algorithm can be used to select the features of the existing data and set different warning values for the selected elements. If it does not exceed the warning value, it indicates that the relevant indicators can operate generally within a reasonable range of changes; if this warning value is exceeded, it suggests that there is financial risk. The supply chain financial risk prevention model will give the warning to inform managers that decisions should be suspended to solve the financial risk problems faced promptly and prevent enterprises from expanding losses.

### 3.2. Support Vector Machine Algorithm Structure

Support vector machine (SVM) is a broad linear catalog that performs a duality catalog on data by supervised learning [35]. It applies the hinge loss function to compute the operation experience risks. It includes a holomorphic item to the solution setup, to achieve the purpose of optimizing the structural risk. SVM can use the nonlinear mapping principle: *f* : *R*^*n*^⟶*H*, to shine upon the nonlinear problem in a high-dimensional room, thereby transforming it into a linear problem [36]. The conforming sample data set is *D*={*x*_*i*_, *y*_*i*_}, *i*=1,2,…*n*, *x*_*i*_ ∈ *R*^*n*^, *y*_*i*_ ∈ *R*, where *x*_*i*_ is the afferent feature eigenvector and *y*_*i*_ is the output load value; the SVM algorithm transforms *x*_*i*_ into a high-dimensional room by shining upon *f*(*x*) to the expression:

(15) $$\begin{array}{c} y=\omega *f\left(x\right)+b\text{.} \end{array}$$

In the formula, *ω* is the slope and *b* is the intercept. *x*=(*x*_1_, *x*_2_ ⋯ *x*_*n*_) is the feature vector, and *y*=(*y*_1_, *y*_2_ ⋯ *y*_*n*_) is the output load value.

To find the optimal *ω* and *b*, the expressions need to satisfy the following conditions:

(16) $$\begin{array}{c} \left\{\begin{array}{c} \min\frac{1}{2}{\left\|\omega \right\|}^{2}+C\overset{n}{\underset{i=1}{\sum }}\left(\xi_{i}+\xi_{i}^{*}\right)\text{,}\\ \text{s}.\text{t}.\left\{\begin{array}{c} \omega *\varphi \left(x_{i}\right)-y_{i}+b\leq \varepsilon +\xi_{i}^{*},\\ y_{i}-\omega *\varphi \left(x_{i}\right)-b\leq \varepsilon +\xi_{i},\\ \xi_{i}^{*}\geq 0,\xi_{i}\geq 0,i=1,2,\cdots n. \end{array}\right. \end{array}\right. \end{array}$$

In the formula, *C* is the punishment term, *ξ*_*i*_ and *ξ*_*i*_^*∗*^ are the relaxation term, having some error, and *ε* is the most significant deviation, which is the parameter of the linear insensitive loss function.

To enhance the overall optimization effect of the model, the Lagrange multiplier method can translate it into a twofold matter:

(17) $$\begin{array}{c} \left\{\begin{array}{c} \min\frac{1}{2}\overset{n}{\underset{i=1,j=1}{\sum }}\left(a_{i}^{*}-a_{i}\right)\left(a_{j}^{*}-a_{j}\right)K\left(x_{i},x_{j}\right)+\varepsilon \overset{n}{\underset{i=1}{\sum }}\left(a_{i}^{*}+a_{i}\right)-\\ \overset{n}{\underset{i=1}{\sum }}y_{i}\left(a_{i}^{*}-a_{i}\right),\\ \text{s}.\text{t}.\left\{\begin{array}{c} \overset{n}{\underset{i=1}{\sum }}\left({a_{i}-a}_{i}^{*}\right)=0,\\ 0\leq a_{i}^{*},a_{i}\leq C,i=1,2,\cdots n. \end{array}\right.\; \end{array}\right. \end{array}$$

In the formula, *a*_*i*_ and *a*_*i*_^*∗*^ are Lagrange multipliers, *K*(*x*_*i*_, *x*_*j*_) is the kernel gamma, and its expression is

(18) $$\begin{array}{c} K\left(x_{i},x_{j}\right)=\exp\;\left(-\gamma {\left\|x_{i}-x_{j}\right\|}^{2}\right)\text{.} \end{array}$$

In the formula, *γ* is the kernel parameter.

So the final expression is

(19) $$\begin{array}{c} y=\overset{n}{\underset{i=1}{\sum }}\left({a_{i}-a}_{i}^{*}\right)K\left(x_{i},x_{j}\right)+b\text{.} \end{array}$$

**Figure 3:**
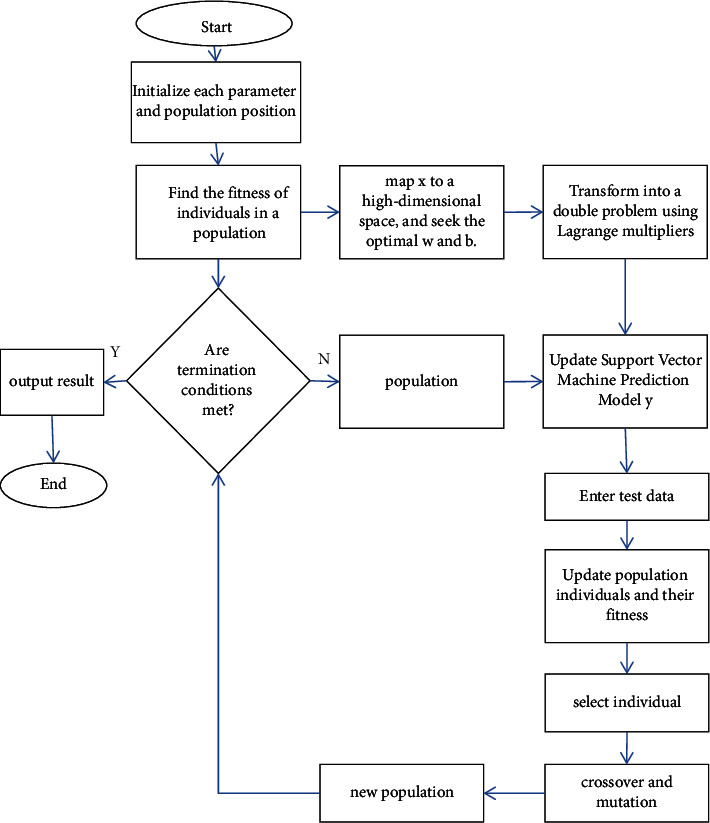
Execution process of SVM.

In the support vector machine (SVM) algorithm, the punishment term *C* and the kernel parameter *γ* are momentous throughout the whole course. *C* reflects the generalization ability and error size of the pattern. The more significant the C is, the smaller the effect of the pattern. However, the smaller the C is, the smaller the generalization ability of the pattern. *γ* will affect the fit of the model. To enhance the model's accuracy, it is requisite to find a better combination of (*C*, *γ*), so it needs to be further optimized. The calculation flow of the Support Vector Machine is shown in Figure 3.

The SVM algorithm can categorize the specimen data after feature selection. Based on different early warning indicators, different risk levels can be classified. The results after classification are helpful for supply chain managers to timely perceive the current operating conditions and take corresponding measures to prevent supply chain financial risks.

### 3.3. Support Vector Machine Algorithm Based on SMA Optimization

Among the swarm intelligence algorithms, the slime mould algorithm (SMA) was put forward by Li et al. [37] and other scholars in 2020. It mainly simulates the spread activity and looks for the food behavior of slime molds [38]. The adaptive weight affects the propagation wave of slime molds on the basis of organism oscillators, thereby generating positive and inverse feedbacks, which is an optimal connection path with better exploration ability and development tendency [39]. The realization process is mainly split into the following measures.

Firstly, initializing parameters and the position of a population, wherein the population satisfies the characteristics of randomly distributed search individuals in a search space, the formula is

(20) $$\begin{array}{c} \overset{⟶}{X\left(t+1\right)}=\left\{\begin{array}{cc} \overset{⟶}{X_{b}\left(t\right)}+\overset{⟶}{vb}*\left[\overset{⟶}{W}*\overset{⟶}{X_{A}\left(t\right)}-\overset{⟶}{X_{B}\left(t\right)}\right]\text{,} & r<p\text{,}\\ \overset{⟶}{vc}*\overset{⟶}{X\left(t\right)}\text{,} & r\geq p\text{,} \end{array}\right.\\ p=\tan\;h\left|S\left(i\right)-DF\right|\text{.} \end{array}$$

Medium formula, $\overset{⟶}{X}$ means the location of the slime mold, *t* means the present iteration times, $\overset{⟶}{X_{A}}$ and $\overset{⟶}{X_{B}}$ represent the individuals randomly selected from the slime mold, respectively, tan *h* represents the hyperbolic tangent function, and $\overset{⟶}{W}$ represents the probability of slime mold, $\overset{⟶}{vb}$ and $\overset{⟶}{vc}$ both represent parameters that meet a specific range, and the expression is

(21) $$\begin{array}{c} \begin{array}{c} \overset{⟶}{vb}=\left[-a,a\right],\overset{⟶}{vc}=\left[-b,b\right]\text{,}\\ a=artanh\left[-\;\left(\frac{T}{\mathrm{Max}\_t}\right)+1\right],\\ ab=1-\left(\frac{T}{Max_{t}}\right)\text{.} \end{array} \end{array}$$

In the formulation, *T* is the time of iteration at present, Max_*t* represents the most considerable iteration times, and artan*h* represents the inverse hyperbolic function.

In the second phase, the adaptive value of apiece search individual is counted, and then the search individuals are sorted, and the weight $\overset{⟶}{W}$ is calculated as

(22) $$\begin{array}{c} \overset{⟶}{W}\left(I\left(i\right),j\right)=\left\{\begin{array}{cc} 1+r*\;\log\;\left(\frac{bF-S\left(i\right)}{bF-wF}+1\right)\text{,} & i\leq \frac{N}{2}\text{,}\\ 1-r*\;\log\;\left(\frac{bF-S\left(i\right)}{bF-wF}+1\right)\text{,} & i>\frac{N}{2}\text{,} \end{array}\right.\\ sort\left(F\right)=\left[S,I\right]\text{.} \end{array}$$

In the formula, *i*=1,2, ⋯*N*, *j*=1,2, ⋯*D*, *N* is the population number, and *D* is the dimension. *r* represents a stochastic digit in the domain of [0, 1], and *bF* and *wF* represent the first-rate fitness and the worst adaptation embodied in the current iteration process, individually. sort(*F*) means the result of sorting the fitness values.

The third step is to find the overall situation's best place and fitness value. To get the individual's current optimal position $\overset{⟶}{X}$, continuously adjust the parameters $\overset{⟶}{vb}$ and $\overset{⟶}{vc}$, and the weight $\overset{⟶}{W}$, draw the individual's activity trajectory in the three-dimensional space, and seek out the best consequence, the expression formula is

(23) $$\begin{array}{c} X_{i}=rand\left(UB-LB\right)+LB\text{.} \end{array}$$

In the formula, *X*_*i*_ means the *i*-th search individually, rand refers to a random digit between the values [0,1], and the *UB* and *LB* distributions embody the superjacent and nether demarcations in the seek area.

The fourth step is to update the individual location. After adjusting the parameters $\overset{⟶}{vb}$ and $\overset{⟶}{vc}$ and the weight $\overset{⟶}{W}$, the location expression of the scouted individual operation is updated as

(24) $$\begin{array}{c} \overset{⟶}{X^{*}}=\left\{\begin{array}{cc} rand\left(UB-LB\right)+LB\text{,} & rand<z\text{,}\\ \overset{⟶}{X_{b}\left(t\right)}+\overset{⟶}{vb}*\left[\overset{⟶}{W}*\overset{⟶}{X_{A}\left(t\right)}-\overset{⟶}{X_{B}\left(t\right)}\right]\text{,} & r<p\text{,}\\ \overset{⟶}{vc}*\overset{⟶}{X\left(t\right)}\text{,} & r\geq p\text{.} \end{array}\right. \end{array}$$

In the formula, both rand and *r* represent random values between [0, 1].

**Figure 4:**
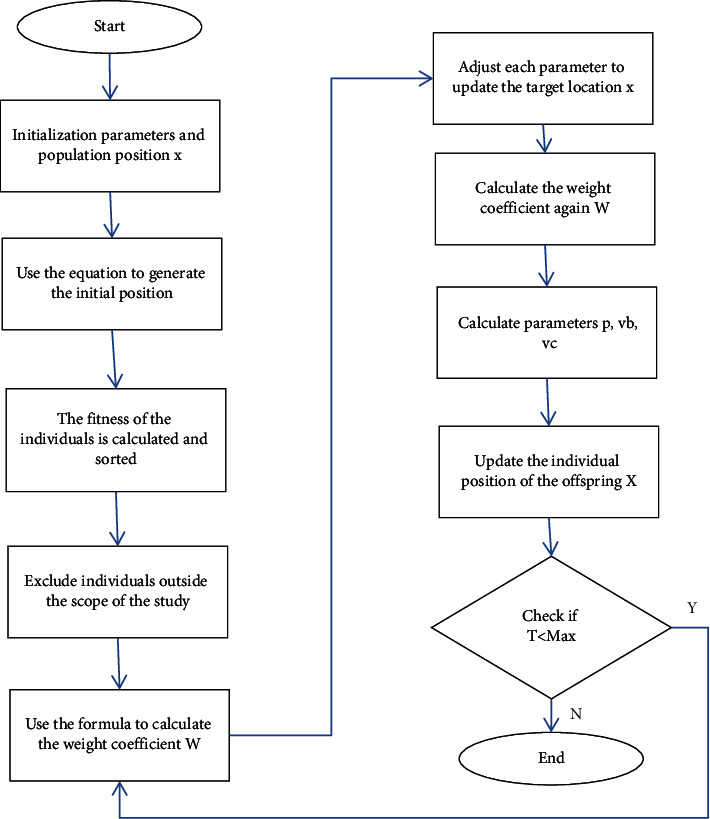
Operational process of SMA.

The overall operation course of the slime mould algorithm is revealed in Figure 4.

To enhance the classification efficiency of the support vector machine algorithm and explore the potential development ability of SVM, this paper proposes an optimized support vector machine algorithm based on the slime mould algorithm (SVM-SMA). The foraging algorithm adjusts and continuously optimizes the relevant parameters to enhance the rationality of the final decision.

In the SVM-SMA model, through the optimization of the slime mould algorithm, the situation of the searched private ownership is adjusted, and the support vector machine algorithm is used to calculate the fitness of the searched individual, and the following parameters are used for classification, to achieve the purpose of optimizing SVM:

(25) $$\begin{array}{c} ada\;ptability=\frac{\sum_{i=1}^{K}{acc}_{i}}{K}\text{.} \end{array}$$

**Figure 5:**
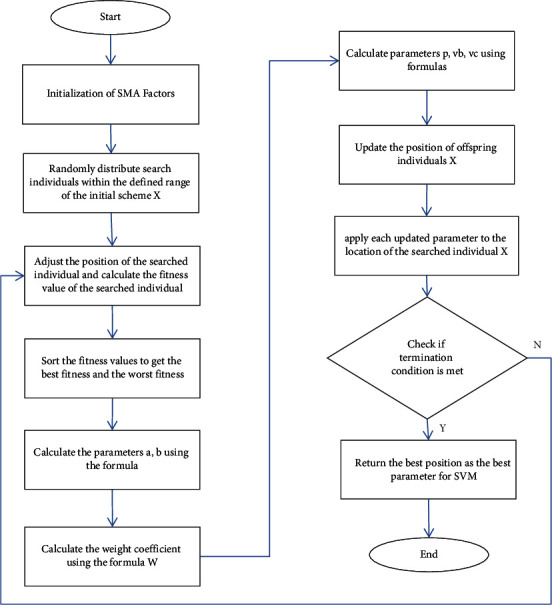
Execution process of SVM-SMA.

To sum up, the execution flow of SVM-SMA proposed in this paper is revealed in Figure 5.

## 4. Numerical Examples

In this paper, all A-share listed companies of China Shenzhen Stock Exchange and Shanghai Stock Exchange in recent three years are selected as research samples. The data are from stock exchanges. Firstly, the data are preprocessed, and the screening process includes the following procedures: (1) eliminating companies with a shortcoming or exceptional data; (2) standardization processing of sample data; (3) excluding the financial industry. Then, the panel data are analyzed with STATA13.0, and all continuous variables are subjected to 1% up and down Winsorize to overcome the influence of extreme values on the experimental results.

Python is used to model the preprocessed data. Firstly, the chaotic locust optimization algorithm is used to select the features. After selecting the elements, the range of the data is simply analyzed. Descriptive index data are expressed on each information in the model. The consequences are displayed in Table 1.

From the data index, we can discover that the amount of companies increases gradually over the years, showing an increasing trend. Each characteristic value will also have a slight change in different years, but the difference is not significant and is relatively stable as a whole. Each characteristic of the same year will be other, and each character is representative to a certain extent.

In view of the existing research, this paper constructs a confusion matrix to examine the effect of the decision of strategic importance of the pattern [40], examine the relationship between the actual results and the decision-making results, and explore the decision-making accuracy of the pattern. The confusion matrix is displayed in Table 2.

If the actual result is normal and the decision result is normal, then TP; if the true result is normal and the decision result is abnormal, FN; if the true result is abnormal, and the decision result is normal, FP; if the true result is abnormal and the decision result is abnormal, it is TN. TN and TP represent the number of correct decisions made by abnormal samples and normal samples, respectively. The higher the value is, the better the decision. FN and FP represent the number of erroneous decisions with abnormal samples and those with normal samples, respectively. A smaller value is better, indicating a smaller error.

Due to the extensive data of the sample companies selected in this paper, the decision-making results cannot be seen more intuitively only based on the confusion matrix. Therefore, relevant evaluation indicators are constructed on this basis, and the model results are evaluated through the calculation of indicators. The specific indicators are shown in Table 3.

In this paper, the CGOA is applied to feature selection, the SVM is used to classify the data after feature selection, the SMA is used to adjust the parameters to seek out the best solution, and the financial risk standard of the supply chain is evaluated. The average value of each year is calculated based on the results, and the results are compared with the decision tree model and the BP-NN model. The decision consequences are displayed in Table 4.

From the results, for overall accuracy, the model CGOA-SVM-SMA proposed in this paper has a better decision-making effect. In the general sample, the proportion of correct decision-making is 85.38%, which is higher than the comparison of the two models. For F-Score and TNR, which are two harmonic averages, the accuracy rate has decreased, because the sample companies have different accuracy rates in normal samples and abnormal samples. However, in general, CGOA-SVM-SMA is better than else patterns in three aspects of PRE, F-Score, and TNR and has higher accuracy. The application of the pattern in financial risk prevention of enterprises has specific practical value.

## 5. Conclusion

Today's Internet technology has become more and more popular, and the Internet financial risks of supply chains can be seen everywhere, bringing enormous challenges to the development of enterprises. In order to help supply chain enterprises make better decisions and deal with the financial risks faced by enterprises, this paper proposes a supply chain digital financial risk prevention model, which mainly includes the pretreatment stage, the feature selection stage based on CGOA, the data classification based on SVM, and the parameter optimization process based on SMA. Preprocessing the data in the light of collecting the data to improve information quality; in the feature selection stage, the chaotic grasshopper optimization algorithm is further optimized based on the grasshopper optimization algorithm, and the preprocessed data indexes are selected for feature selection, which paves the way for the following research; the robust support vector machine algorithm is used to classify the data indexes. Based on that, the slime mold foraging optimization algorithm optimizes the parameters to find the best solution for the decision system. Using this model to analyze the relevant data in the recent three years, it is found that the simulation results obtained are effective in financial performance, accuracy, sensitivity, and other aspects and have a good application prospect, which can effectively and accurately help supply chain enterprises to prevent financial risks. Through the model validation of the sample data of all A-share public corporations in China Shenzhen Stock Exchange and Shanghai Stock Exchange, it is found that the model can well prevent financial risks and enhance the financial performance result of the supply chain. The accuracy rates of the data model can reach 85.38%, respectively, after the test, indicating that the model has good prediction and decision-making effects and can enhance the reliability and reliability of the decision-making results. The main body of the supply chain can discover the financial risks faced by the enterprises in time with the aid of the model, analyze the risk levels faced by the enterprises, and take corresponding measures to prevent the financial risks in the supply chain in time. The limitation of this research lies in that the influence of outliers is not considered, the companies with the abnormal operation and missing data are removed in the data preprocessing process, and only representative extensive sample data are selected. In future research, the method of outlier detection can be added to the model CGOA-SVM-SMA or the model can be improved to enhance further the precision of the supply chain financial risk prevention pattern.

## Figures and Tables

**Table 1:** Descriptive statistics.

	Category	Amount	Minimum	Maximum	Average
The first year	Feature 1	9764	−1.3714	0.5278	0.0616
	Feature 2	9764	−1.3628	0.5261	0.0479
	Feature 3	9764	−0.6670	3.5698	0.1326
	Feature 4	9764	0.0322	5.2401	1.1368

The second year	Feature 1	10596	−0.8654	0.6658	0.06688
	Feature 2	10596	−0.8730	0.5412	0.0525
	Feature 3	10596	−0.7888	8.8841	0.1863
	Feature 4	10596	0.0143	0.9607	0.3799

The third year	Feature 1	11960	−0.3733	0.7093	0.07015
	Feature 2	11960	−0.3949	0.6042	0.0574
	Feature 3	11960	−0.5429	3.9448	0.2203
	Feature 4	11960	0.0189	0.9032	0.3746

**Table 2:** Financial risk confusion matrix.

		Decision results	
		Abnormal	Normal
Real results	Abnormal	TN	FP
	Normal	FN	TP

**Table 3:** Evaluation indicators.

name	Formula	Meaning
PRE	PRE=TP+TN/TP+TN+FP+FN	Number of sample proportions with correct decisions
REC	REC=TP/TP+FN	The number of correctly predicted proportions in the normal sample
F-score	(*α*^2^+1) × PRE × REC/*α*^2^ × PRE+REC	Harmonic mean value of PRE and REC (*α* = 1 in this paper)
TNR	$TNR=\sqrt{TP/TP+FN\times TN/TN+FP}$	Average predictive accuracy of normal and abnormal samples

**Table 4:** Comparative analysis of decision results.

Model	PRE	F-score	TNR
DT	0.57	0.38	0.65
BP-NN	0.71	0.45	0.67
CGOA-SVM-SMA	0.85	0.63	0.72

## Data Availability

The data used to support the findings of this study are available from the corresponding author upon request.
